# Directed percolation identified as equilibrium pre-transition towards non-equilibrium arrested gel states

**DOI:** 10.1038/ncomms11817

**Published:** 2016-06-09

**Authors:** M. Kohl, R. F. Capellmann, M. Laurati, S. U. Egelhaaf, M. Schmiedeberg

**Affiliations:** 1 Institute for Theoretical Physics II: Soft Matter, Heinrich Heine University, Universitätsstraße 1, 40225 Düsseldorf, Germany; 2 Condensed Matter Physics Laboratory, Heinrich Heine University, Universitätsstraße 1, 40225 Düsseldorf, Germany; 3 Institute of Theoretical Physics 1, Friedrich-Alexander University Erlangen-Nürnberg, Staudtstr. 7, 91058 Erlangen, Germany

## Abstract

The macroscopic properties of gels arise from their slow dynamics and load-bearing network structure, which are exploited by nature and in numerous industrial products. However, a link between these structural and dynamical properties has remained elusive. Here we present confocal microscopy experiments and simulations of gel-forming colloid–polymer mixtures. They reveal that gel formation is preceded by continuous and directed percolation. Both transitions lead to system-spanning networks, but only directed percolation results in extremely slow dynamics, ageing and a shrinking of the gel that resembles synaeresis. Therefore, dynamical arrest in gels is found to be linked to a structural transition, namely directed percolation, which is quantitatively associated with the mean number of bonded neighbours. Directed percolation denotes a universality class of transitions. Our study hence connects gel formation to a well-developed theoretical framework, which now can be exploited to achieve a detailed understanding of arrested gels.

## Abstract (web summary)

Gels exhibit very slow dynamics, for which a structural reason remains elusive. Here, Kohl *et al*. show the gel formation is accompanied by a succession of continuous and directed percolation, with only the latter found to lead to the arrested dynamics.

## Graphical abstract

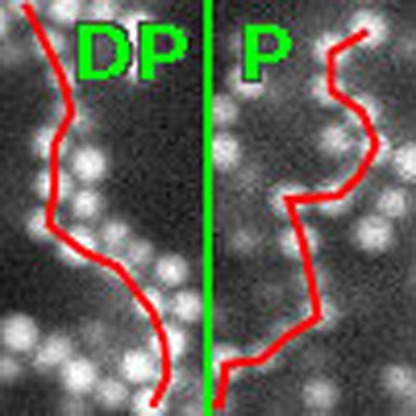

Amorphous solids are ubiquitous in natural and engineered materials: sand piles, window glass, ceramics, plastics, nanocomposites, gelatin and living cells are just a few examples of solid materials with an amorphous structure. Glassy systems are amorphous solids in which the solid-like mechanical properties are linked to a dramatic slowdown of the microscopic dynamics when decreasing the temperature or increasing the particle density^1^,^2^,^3^,^4^,^5^,^6^,^7^. Gels also show arrested dynamics^8^,^9^,^10^,^11^,^12^,^13^,^14^,^15^,^16^,^17^, but their structure is different. They exhibit a heterogeneous load-bearing network structure that is formed by cross-linking (chemical gels) or attraction-induced aggregation (physical gels)^18^,^19^,^20^,^21^,^22^,^23^,^24^,^25^,^26^,^27^,^28^,^29^,^30^,^31^. Dynamical arrest was associated with different mechanisms, for example, arrested phase separation^21^, cluster aggregation^21^, the occurrence and spatial organization of locally favoured structures^18^ and the onset of rigidity percolation^32^. Furthermore, a progressive slowdown of the dynamics after sample preparation, that is, ageing, is observed. However, the relation between the slowdown and arrest of the dynamics, and the structural properties of these systems are still under debate^8^,^9^.

Model systems of physical gels have been established, for example, dispersions of spherical colloidal particles, in which attractive forces are induced by a temperature variation or depletion effects due to the addition of linear non-adsorbing polymer chains^30^,^31^. Nevertheless, there are only few studies on the relation between the slowdown of the dynamics and the microscopic structure.

Here we show that the slowdown of the particle dynamics and the onset of ageing can be related to the microscopic structure of colloidal gels, namely a directed percolation (DP) transition. This is demonstrated in experiments on charged colloid–polymer mixtures^30^,^33^ and in Brownian dynamics simulations of particles interacting through a combination of coulombic repulsion and depletion-induced attraction modelled by the Asakura–Oosawa potential^34^. The onset of DP is investigated along different paths in which either the attractive or the repulsive parts of the interactions are varied. We find that in any case continuous percolation precedes DP and that both can be associated with characteristic values of the mean number of bonds per particle. Furthermore, DP is found to be linked to the onset of ageing effects.

## Results

### States of the system

In charged colloid–polymer mixtures, two particle–particle interactions compete on different length scales: First, screened electrostatic interactions, which are long ranged for low salt concentrations and become shorter ranged on addition of salt; second, depletion interactions, which are short ranged, typically at most one tenth of the colloidal particle diameter, and controlled through the radius of gyration of the polymers. We experimentally investigated charged colloidal polymethylmethacrylate (PMMA) spheres with volume fraction *Φ*≈0.2 and diameter *σ*=1.72 μm in the presence of non-adsorbing linear polystyrene with radius of gyration *r*_g_=65 nm, where the effective polymer-colloid size ratio *ξ*^eff^≈0.03 (Supplementary Table 1).

**Figure 1:**
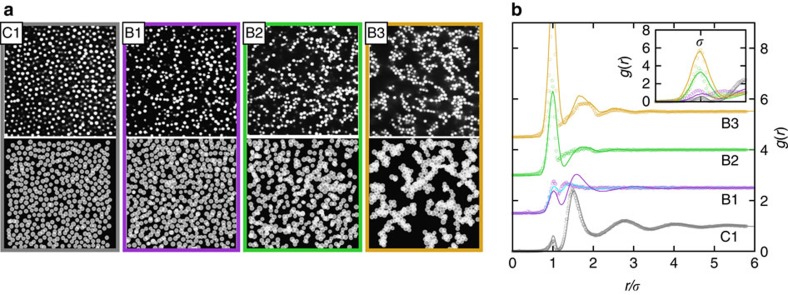
On the path towards a gel four different states are found. (**a**) Two-dimensional slices of the three-dimensional systems in the following states: (C1) Fluid state with mainly individual particles and only very few small aggregates, (B1) isolated small chain-like clusters of particles, (B2) larger clusters forming a continuously percolated network in three dimensions and (B3) directed percolated network. The upper row shows confocal microscopy data, where the polymer concentrations are 3c* (C1) and 2c* (B1–B3) with the overlap concentration c*. The salt concentration increases from left to right (Supplementary Table 1). The lower row represents the corresponding states as observed in the simulations. (**b**) Corresponding pair correlation functions *g*(*r*) determined from the three-dimensional data as a function of the particle-particle distance *r* in units of the particle diameter σ, which, for the simulation data, is taken to be the effective particle diameter, that is, *σ*=*σ*^eff^. Symbols represent experimental data, solid lines simulation data and (only for B1) the light blue solid line simulation data with parameters obtained by an unconstrained fit (for details see Supplementary Note 1). Data have been shifted for clarity. The inset shows the (unshifted) first peaks.

In experiments and simulations, we consistently observe four states that differ in the structural arrangements of the colloidal particles (Fig. 1, left). Which state occurs depends on the salt concentration *c*_salt_, and therefore the screening length *κ*^−1^ characterizing the range of the repulsion and the polymer concentration *c*_p_, that is, attraction strength. We consider fixed *c*_p_ and vary *c*_salt_, which will be referred to as ‘path B'. In the absence of salt, mainly individual particles but also very few clusters are distributed homogeneously throughout the sample (sample C1; note that *c*_p_ of this sample is higher than the one of the other samples, Supplementary Table 1). Adding salt, particles aggregate into isolated small clusters (sample B1). At large salt concentrations, larger clusters are observed which form a network in three dimensions (sample B2). Furthermore, a heterogeneous network structure with thick strands consisting of even larger, dense clusters are formed at the largest salt concentration studied (sample B3).

These states show different pair correlation functions *g*(*r*) (Fig. 1, right). Without salt (C1), *g*(*r*) is dominated by a peak located at the mean particle distance *ρ*^−1/3^. Furthermore, a small peak at particle contact reflects the presence of a small number of aggregated particles. For small amounts of added salt (B1), the increase in the contact peak and a corresponding decrease of the second peak of *g*(*r*) is consistent with the presence of doublets, triplets or small string-like clusters (visible in Fig. 1a). The size of the clusters is limited by the repulsive contribution to the potential^22^,^35^,^36^. For sample B2, the large peak at contact reflects the large fraction of particles forming clusters. Its *g*(*r*) is reminiscent of a percolated gel-like network structure^32^,^37^,^38^. Finally, the radial distribution function of sample B3 with its very high first peak at contact reflects the presence of a large number of bonded particles. Furthermore, its deep first minimum and pronounced split second peak can arise from triplet structures and local close packed arrangements. These features of the *g*(*r*) are observed in the experiments as well as in the simulations. While there is qualitative agreement between experimental and simulation results, small quantitative differences indicate that the potential and its parameter values used in the simulations are not perfectly describing the interactions present in the samples. (The mapping of the simulation and experimental parameters is described in Supplementary Note 1.) Nevertheless, both, experiments and simulations, reveal different states on the path towards a gel; homogeneously distributed individual particles, small clusters, networks with thin and thicker strands consisting of larger, more compact and denser clusters, respectively.

### Characterization of the different states by number of bonds

The different structures are distinguished by the degree of particle aggregation. This can be linked to the mean number of bonds per particle, 〈*N*〉. Individual particle–particle bonds cannot be determined unambiguously because the first peak in *g*(*r*) is broadened due to polydispersity (about 7%). Hence, two particles are defined to be bonded if they are closer than the position of the first minimum in *g*(*r*), which is located between 1.10 and 1.22*σ* (Fig. 1, right).

**Figure 2:**
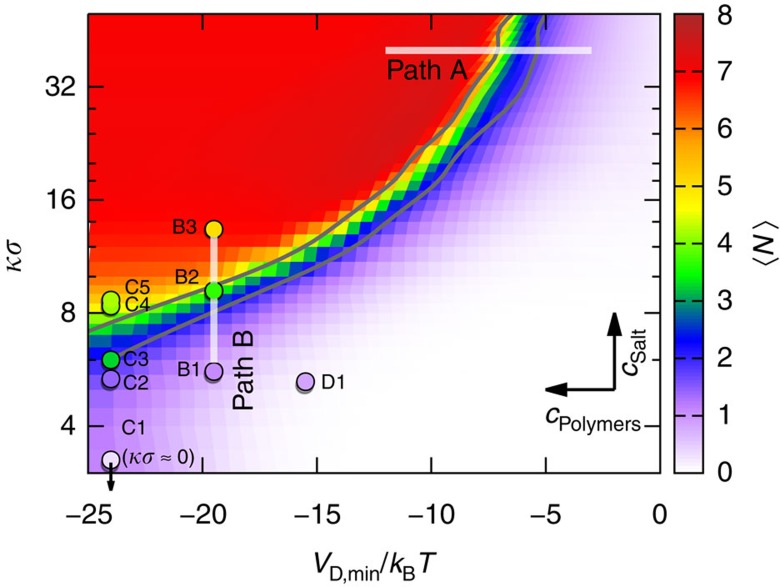
The mean number of bonds characterizes the different stages towards a gel. Mean number of bonds per particle, 〈*N*〉, as a function of the minimum of the attractive part of the potential, evaluated at the global minimum, *V*_D,min_ in units of the thermal energy *k*_B_*T*, and the inverse screening length *κ* normalized by the inverse particle diameter *σ*^−1^, which, for the simulation data, is taken to be the mean particle diameter, that is, *σ*=*σ*_0_, in a semi-logarithmic representation. The background colour represents 〈*N*〉 as obtained by simulations, while the positions and colours of the circles indicate the compositions and 〈*N*〉, respectively, of the experimental samples, which are labelled. The two white lines indicate two paths, during which either only the polymer concentration (‘path A') or only the salt concentration (‘path B') are changed. The lower and upper dark solid lines indicate the continuous and directed percolation transitions, respectively, which are taken to occur when the corresponding *p*(*l*_box_)=0.2 (Fig. 4c).

The mean number of bonds per particle, 〈*N*〉, is determined as a function of the interactions, which depend on the salt concentration *c*_salt_, determining the screening length *κ*^−1^, and the polymer concentration *c*_p_, controlling the depletion part of the potential *V*_D,min_=*V*_D_(*σ*^eff^) evaluated at the overall potential minimum *V*_min_=*V*(*σ*^eff^) (Fig. 2). At low polymer, *c*_p_, and/or salt, *c*_salt_, concentrations, only very few bonds are found (white to purple), whereas high *c*_p_ and/or *c*_salt_ result in a bonded state (red). In contrast to previous results^23^, 〈*N*〉 monotonically grows without a re-entrant transition. Even for large screening and strong attraction, 〈*N*〉 hardly exceeds seven and hence the highly bonded state appears not to be in equilibrium. For a purely repulsive interaction^39^,^40^ or in spring lattice models^41^ such a change is expected once the system becomes isostatic, that is, globally stable with six bonds per particle.

**Figure 3:**
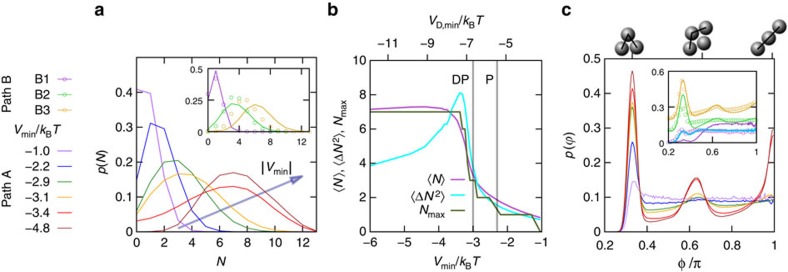
Quantitative analysis of structures along paths from fluid to gel. (**a**) Distribution of the number of bonds per particle, *p*(*N*), as obtained by simulations for constant screening length *κσ*=40 and varying attraction (as indicated on the left), that is, path A in Fig. 2. In the inset simulation (lines) and experimental (circles) results for varying *κσ* and constant attraction, that is, path B, are compared. Along the paths, *p*(*N*) first broadens, revealing a coarsening of the structure, before narrowing again (along path A). This indicates rather homogeneous structures for high attractions. (**b**) Mean 〈*N*〉, variance 〈Δ*N*^2^〉 and the most probable value *N*_max_ of *p*(*N*) as a function of attraction strength, *V*_min_, along path A as obtained by simulations. All three functions show an increase at *V*_min_≈−3.0 *k*_B_*T*. The grey vertical lines denote the percolation (P) and the directed percolation (DP) transition as determined through the condition *p*(*l*_box_)=0.2 (Fig. 4c). (**c**) Distribution of the angle between two successive bonds, 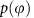, along path A as obtained by simulations. The inset shows 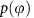 along path B as obtained by simulations (lines) and experiments (circles). Also 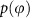 reveals a qualitative change at *V*_min_≈−3.0 *k*_B_*T*, as the peaks at 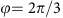 and 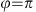 become pronounced. This reflects the occurrence of straight thick string-like particle formations. The light blue line in the inset represents simulation data with parameters obtained by an unconstrained fit (for details see Supplementary Notes 1). Colours of the other lines as in **a** and indicated on the left.

The structures along two paths are analysed in more detail (Fig. 3). Along path A, the salt concentration *c*_salt_ and hence the screening length *κ*^−1^ is kept constant while the polymer concentration *c*_p_ is increased and hence the value *V*_D,min_ of the potential at its minimum is decreased, that is, the attraction strength is increased. (Note that path A implies a high salt concentration, which cannot be achieved in the organic solvent mixture used in the experiments.) Instead, along path B, the screening length *κ*^−1^ is reduced at constant attraction strength. On increasing attraction on path A, 〈*N*〉 increases and the distribution of the number of bonds per particle, *p*(*N*), first broadens, indicating a more heterogeneous structure, but then narrows again indicating that the probability to find monomers is reduced to zero, consistent with the observed network structure (Figs 1a,3a). Along path B, that is, increasing *c*_salt_ (inset), the distributions also shift to larger *N* and broaden, again indicating the coarsening of the structure. Compared with path A, the *p*(*N*) are sharper, indicating that monomers are suppressed at low salt concentrations. Under these conditions, repulsions are long ranged and still substantial at the mean monomer separation. The system avoids this unfavourable situation by forming clusters. Although the repulsion between the clustered monomers is even larger, cluster formation is favoured by the short-ranged attractions and the larger mean cluster separation that reduces cluster–cluster repulsion.

We characterize *p*(*N*) by its mean 〈*N*〉, variance 〈Δ*N*^2^〉 and maximum *N*_max_. With increasing attraction, that is, |*V*_D,min_|, they all sharply increase at *V*_min_≈−3.0 *k*_B_*T*, where *k*_B_*T* is the thermal energy (Fig. 3b). Furthermore, we consider the distribution 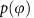 of angles between two successive bonds, 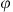. The distribution is normalized by the solid angle covered by the angle 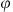, that is, such that 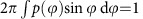. It reveals a qualitative change that also occurs at *V*_min_≈−3.0 *k*_B_*T* (Fig. 3c). While the peak at 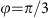, which is related to locally dense tetrahedral packings, grows monotonically with |*V*_min_|, a second peak at 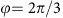 develops and becomes pronounced for 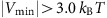. Furthermore, for these large potential depths, a peak at 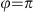 emerges, which indicates the formation of straight strings of connected particles. Therefore, based on the mean number of bonds per particle 〈*N*〉, the different structures on the path towards a gel can be distinguished and quantitatively characterized.

### Continuous and DP transitions

**Figure 4:**
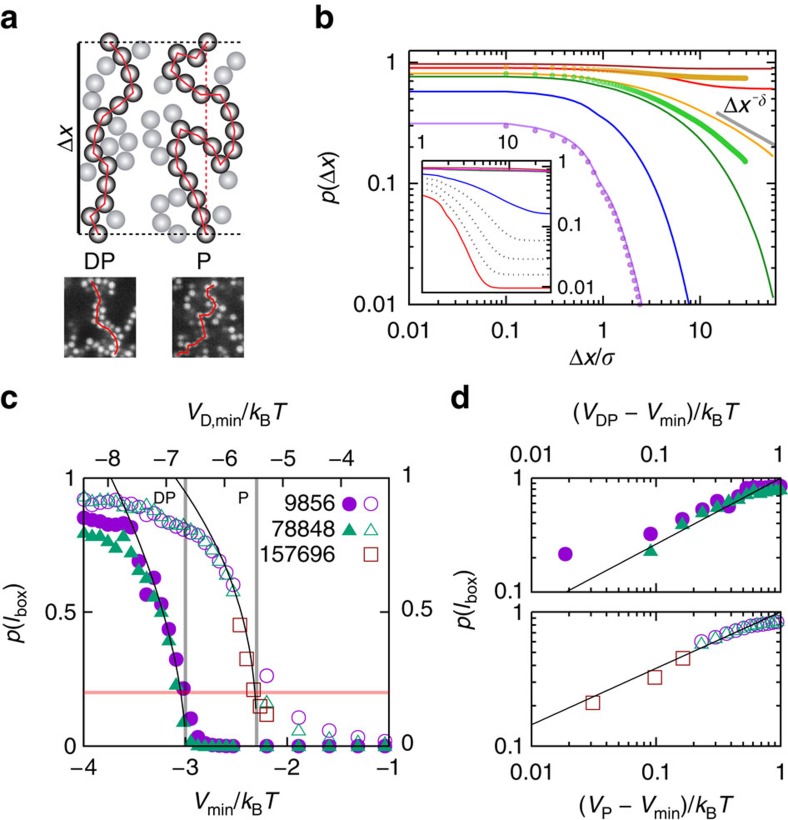
Continuous and directed percolation and their transitions. (**a**) Two-dimensional sketch as well as images from experiments of (DP) a directed and (P) an undirected percolated cluster of length Δ*x*. The path along a directed percolated cluster must not include backward steps in an arbitrarily chosen direction. (**b**) Probability *p*(Δ*x*) for a particle to reside in a (main figure) directed and (inset) continuous cluster with a length larger than the minimum length Δ*x*. Solid lines represent simulation data along path A and symbols experimental data along path B. Colours of the lines are defined in the legend of Fig. 3. The grey solid line indicates the DP critical point, where the percolation probability is expected to be proportional to Δ*x*^−*δ*^ with *δ*=0.451 (ref. 44). (**c**) Probability *p*(*l*_box_) for a particle to reside in a (solid symbols) directed and (open symbols) continuous cluster with the maximum possible length, that is, box size *l*_box_, and hence system-spanning, for different system sizes, quantified by the number of particles *M* (as indicated). Vertical lines indicate the (left) directed and (right) continuous percolation transitions defined by *p*(*l*_box_)=0.2 (horizontal red line) occurring at *V*_DP_=−3.0 *k*_B_*T* and *V*_P_=−2.3 *k*_B_*T*, respectively. Solid black lines represent critical power law fits with (left) *p*(*l*_box_)∼(*V*_DP_−*V*_min_)^0.58^ and (right) *p*(*l*_box_)∼(*V*_P_−*V*_min_)^0.42^. (**d**) Same data as in **c**, but in double-logarithmic representations and relative to the (top) directed and (bottom) continuous percolation transitions.

We will now show that the particle networks undergo two percolation transitions, a continuous percolation transition and a DP transition. In both cases, percolated networks span the whole system. In continuous percolation, a path along a percolating cluster may contain steps in all directions, including backward steps (Fig. 4a, P). Instead, in the case of DP, only paths along an arbitrarily chosen direction are considered whereas possible backward steps in this direction are not considered (Fig. 4a, DP). In the limit of an infinite system size, percolating clusters occur in all directions in the sample. Therefore, on a macroscopic scale no preferred direction exists in a directed percolated network.

The (cumulative) probabilities *p*(Δ*x*) that a particle participates in paths with a projected length of at least Δ*x* and in directed paths with a length of at least Δ*x* along any arbitrarily chosen direction are shown in the inset and main part of Fig. 4b, respectively, where the solid lines represent simulation data along path A and symbols indicate results from experiments along path B. For both percolation conditions, the probabilities decay to zero in the case of weak attractions (small |*V*_min_|). In contrast, for strong attractions plateaus develop, indicating large clusters that span the whole system. The height of the plateaus correspond to the fractions *p*(*l*_box_) of particles that are part of clusters with the maximum possible length, that is, the box size *l*_box_ (Fig. 4c). The system size determines *l*_box_ and also affects the simulations. Nevertheless, within the investigated system sizes only a weak dependence on system size, quantified by the total number of particles *M*, is observed. In particular, the occurrence of the sharp increases of *p*(*l*_box_) and their positions are not significantly affected. These sharp increases indicate the continuous and DP transitions.

We determine the continuous and DP transitions in simulations for the complete parameter space of Fig. 2. The locations are chosen to coincide with the percolation probability *p*(*l*_box_)=0.2 for systems with 9,856 particles (Fig. 4c). Using this condition, along path A, the continuous percolation transition occurs at *V*_P_=−2.3 *k*_B_*T* and the DP transition is found at *V*_DP_=−3.0 *k*_B_*T*. The latter coincides with the significant changes observed in other parameters; 〈*N*〉 (Fig. 2, dark solid lines) as well as *p*(*N*), 〈Δ*N*^2^〉, *N*_max_ and 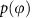 (Fig. 3). Furthermore, this indicates that the continuous percolation transition occurs at 〈*N*〉≈2 and the DP transition at 〈*N*〉≈3 (Fig. 3b). This suggests a connection between the percolation transitions and 〈*N*〉. Previous work^32^,^37^ on particles with attractive square-well interactions also observed continuous percolation for 〈*N*〉≈2. This work moreover suggested that rigidity percolation is associated with gelation, which was found to occur at 〈*N*〉≈2.4, that is, just in between our estimates for the onsets of continuous and DP. The possible link between rigidity and DP deserves further investigation.

Furthermore, close to the transitions the values of *p*(*l*_box_) obey critical power law scaling with *V*_P_−*V*_min_ and *V*_DP_−*V*_min_, respectively (Fig. 4c,d). Note that up to leading order the probability for a bond between neighbours is proportional to *V*_min_ and therefore scaling laws for *p*(*l*_box_) as a function of this probability also hold for *p*(*l*_box_) as a function of *V*_min_. Our data are consistent with theoretical predictions for the critical exponents of the power law scalings, that is, *β*_P_=0.42 for continuous percolation^42^,^43^ and *β*_DP_=0.58 for directed percolation^44^ (black lines in Fig. 4c,d; only the intercepts are fitted and the data with almost constant *p*(*l*_box_) at large *V*_P_−*V*_min_ and *V*_DP_−*V*_min_, respectively, are not included in the fits). These findings strongly suggest that, on the path towards a gel, continuous and DP networks occur with well-defined transitions to other states as well as between them. Furthermore, this links the formation of gels to a universality class of transitions, namely DP, and hence a well-developed theoretical framework.

### Onset of slowdown and ageing in directed percolated systems

**Figure 5:**
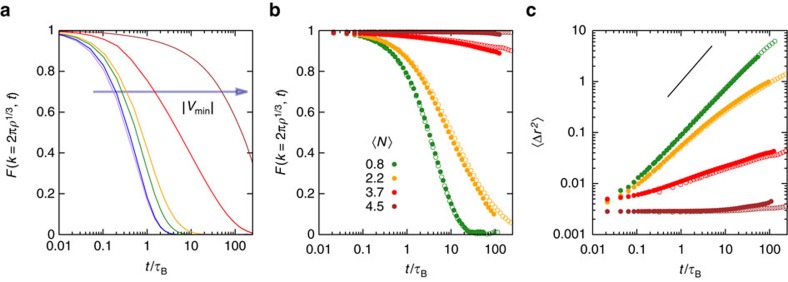
In directed percolated systems the dynamics slow down. Self intermediate scattering function *F* at the scattering vector *k*=2*πρ*^−1/3^ as a function of delay time *t*, normalized by the Brownian time 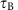, (**a**) along path A as obtained by simulations (with the colours of the lines defined in the legend of Fig. 3) and (**b**) for different mean numbers of bonds 〈*N*〉 (as indicated) as obtained by experiments and (**c**) corresponding mean squared displacements 〈Δ*r*^2^〉 as obtained by experiments. The line indicates a slope of one.

The dynamics of the different structures are quantified by the self-intermediate scattering function *F* and mean squared displacement 〈Δ*r*^2^〉 (Fig. 5). Diffusive dynamics is observed for individual particles and small clusters, characteristic for fluids. In continuous percolated samples, open network structures are formed (Fig. 1, B2) that are rearranged by local motions requiring only slightly more time of the order of the Brownian time 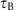 (Fig. 5). In contrast, a marked slowdown of the dynamics is observed beyond the directed percolation (DP) transition. DP is characterized by dense clusters with thick strands (Fig. 1, B3) and thus rearrangements are expected to involve long-range, global motions and the breaking of several bonds. Correspondingly, they require long times. The dynamics of the different states hence are consistent with our structural observations. They suggest that the slowdown associated with gelation sets in at the DP transition and the dynamics eventually arrests deeper in the gel region.

**Figure 6:**
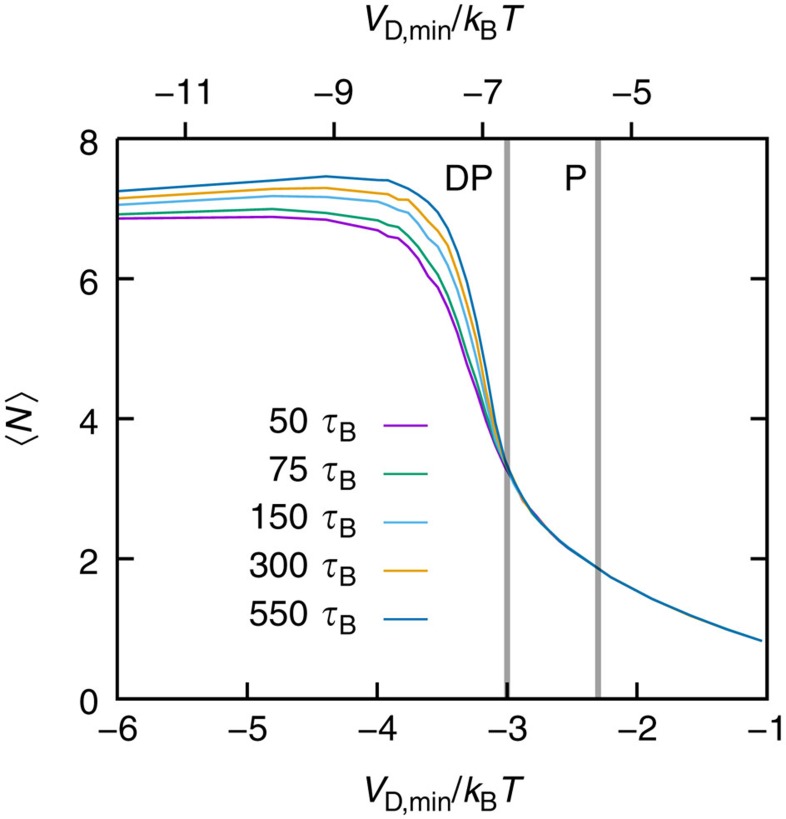
In directed percolated systems ageing occurs. Mean number of bonds per particle, 〈*N*〉, as a function of the attraction strength, *V*_min_, for different waiting times after the initial quench (as indicated, in units of the Brownian time 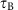) as obtained by simulations. Vertical lines indicate the (DP) directed and (P) continuous percolation transitions as determined through the condition *p*(*l*_box_)=0.2 (Fig. 4c). For systems beyond the directed percolation transition, 〈*N*〉 increases with the waiting time indicating ageing effects.

The simulation data presented so far have been determined for a fixed waiting time of 300 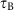 after the initial quench. The effect of the waiting time is illustrated for the dependence of the mean number of bonds per particle, 〈*N*〉, on the potential depth *V*_min_ (Fig. 6), which has been linked to the continuous and DP transitions (Fig. 3b). For small attraction strengths, 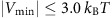, 〈*N*〉 is independent of the waiting time within the examined time range. However, for 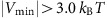 and thus for DP, 〈*N*〉 is found to increase with waiting time. This indicates that ageing effects are important in directed percolated systems and equilibrium is reached only very slowly. In continuous percolation, in contrast, equilibrium is attained quickly. These findings also indicate the importance of the above-mentioned local and global rearrangement processes in continuous and directed percolated systems, respectively. In addition, it also supports the above conclusion that the DP transition is an equilibrium transition just before the onset of dynamical arrest.

### Synaeresis in directed percolated systems

**Figure 7:**
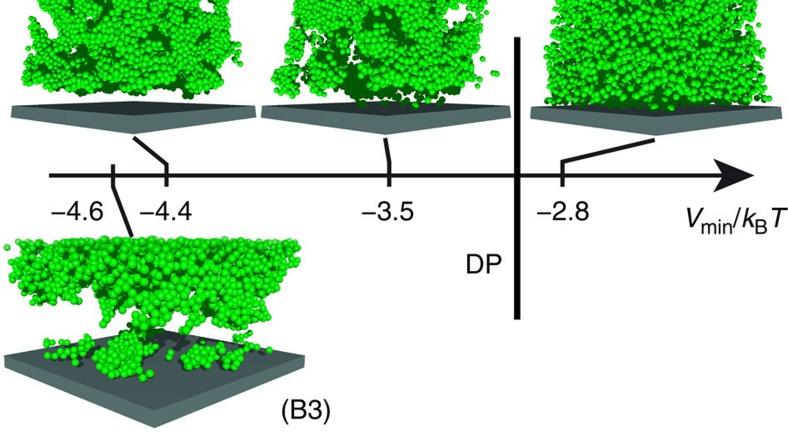
Particle depletion close to walls in directed percolated systems. Rendered snapshots of samples in the vicinity of a wall obtained in (top) simulations with systems along path A and (bottom) an experiment with sample B3. Beyond the directed percolation transition, that is, for *V*_min_/*k*_B_*T*<−3.0, the vicinity of the walls starts to be depleted of particles.

We also investigate the vicinity of walls (Fig. 7). Initially, the particles fill the whole volume, also close to the wall. In fluids and continuously percolated samples this remains like this at least for several weeks (about 10^6^ 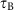). In contrast, in directed percolated systems, the vicinity of the walls becomes depleted of particles in less than a day (about 3 × 10^4^ 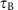), except for very few clusters which remain attached to the cover slip in the experiments, likely due to depletion and van der Waals attractions^45^. The depletion close to the walls indicates that directed connections between particles tend to compact the network. This resembles synaeresis, that is, the macroscopic expulsion of fluid from a gel due to the shrinking of the network. This has been observed in a variety of materials, like gelatin^46^, polysaccharide gels^47^,^48^, protein gels^49^, organogels^50^, microgels^51^ and also weakly attractive colloidal gels^52^. The link between network shrinkage and DP might provide new aspects for the understanding of synaeresis.

## Discussion

We investigate gel formation in a system with competing attractive and repulsive interactions. Confocal microscopy experiments on charged colloid–polymer mixtures are combined with Brownian dynamics simulations of particles interacting via the Asakura–Oosawa and Coulomb potentials. Depending on the overall potential minimum *V*_min_, which can be varied through the attractive and/or repulsive component of the interactions, different states are identified; fluids of individual particles or clusters as well as continuous and directed percolated networks. The transitions between these states are associated with changes in structural and dynamical parameters, in particular, the number of bonds per particle with significant increases in the mean, variance and most probable value of the distribution of the number of bonds.

The effect of continuous percolation on the dynamics is small. However, DP leads to a significant slowdown of the dynamics. This is attributed to the large number of bonds and concomitant strong confinement of the particles in the attractive potentials of their neighbours, which is also reflected in significant ageing observed in directed percolated systems. It suggests that equilibration is very slow in directed percolated systems, but occurs quickly in continuous percolated systems. Hence, our findings suggest that the slowdown associated with gelation already sets in at the DP transition. Deeper in the gel region, the dynamics then becomes arrested without any additional structural transition occurring.

Our finding that DP precedes the formation of arrested gels, establishes a relation between structural and dynamic features of gels. While previous studies linked gelation in adhesive hard spheres to continuous percolation^28^,^29^,^31^, we argue that in colloid–polymer mixtures with competing attractive and repulsive interactions directed rather than continuous percolation indicates the transition to gel states. Moreover, DP is a universality class of transitions. Thus gel formation can be linked to critical behaviour and the corresponding theoretical formalism^53^, which can be exploited to obtain a deeper understanding of gel formation. Moreover, maybe gel collapse under gravity^30^ is also initiated by percolation transitions. Our results hence contribute to an improved understanding of the relation between structural and dynamic features of gel-forming systems and hint at their importance for gel collapse and rheological properties.

Furthermore, in directed percolated systems the dense and directed networks appear to lead to a contraction. This results in a detachment from the walls and hence a layer that is depleted of particles appears. This phenomenon resembles synaeresis and might have important implications for applications and industrial products but also, for example, gel collapse and rheological measurements, in particular concerning wall slip^45^,^54^,^55^,^56^.

## Methods

### Experiments

The samples consist of spherical PMMA particles, sterically stabilized with poly(12-hydroxystearic acid) polymers and fluorescently labelled with 7-nitrobenzo-2-oxa-1, 3-diazole-methyl-methacrylate, as well as linear polystyrene. The particles have a diameter *σ*=1.72 μm and a polydispersity of 7%, which are determined by static and dynamic light scattering of a very dilute suspension (volume fraction *Φ*=0.005). They are dispersed in a mixture of cis-decalin and cycloheptylbromide, which matches the density and almost matches the refractive index of the particles. We do not observe any indication of anisotropy, sedimentation or creaming of the samples during the observation time (days), indicating a high degree of density matching between solvent and particles. In some samples, creaming is observed after several weeks. In this solvent mixture, PMMA particles acquire a charge^33^,^57^.

To prepare the colloid stock solution, the particles are sedimented using a centrifuge. The sediment is assumed to be random close packed with a volume fraction *Φ*_RCP_=0.64 and used to prepare a colloid stock solution with *Φ*≈0.40. The polystyrene has a molar mass *M*_w_=3 × 10^6^ kg mol^−1^ resulting in a radius of gyration *r*_g_=65 nm (ref. 58) and polydispersity characterized by *M*_w_/*M*_n_=1.17. For a dilute mixture, the polymer-colloid size ration 2*r*_g_/*σ*=0.076. Since *r*_g_ depends on concentration, an effective polymer-colloid size ratio *ξ*^eff^≈0.03 is estimated based on the Generalized Free Volume Theory^59^,^60^,^61^ (Supplementary Table 1). Different polymer stock solutions are prepared by dispersing dry polymer in the solvent mixture to yield concentrations 4.3 mg ml^−1^≤*c*_p_=*m*_p_*ρ*_s_/*m*_s_≤18.9 mg ml^−1^ with *m*_p_ the mass of the dry polymer, *ρ*_s_ the density and *m*_s_ the mass of the solvent mixture, respectively. Furthermore, the overlap concentration is calculated using 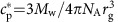 with *N*_A_ Avogadro's constant. To obtain colloid–polymer mixtures with *Φ*≈0.2, colloid and polymer stock solutions are mixed with a volume ratio of 50:50 for samples B1–B3, C1, C3 and D1, while dry polymer is added to the diluted colloid stock solution in the case of samples C2, C4 and C5. (The exact composition of each sample is reported in Supplementary Table 1.) To homogenize the samples, the mixtures are left on a vortex mixer for some minutes and then on a flask shaker for at least one day. To vary the screening length *κ*^−1^, different amounts of salt (tetrabutylammoniumchloride, Supplementary Table 1)^33^ are added and the mixture gently moved on the flask shaker for three days to help the dissolution of the salt in the organic solvent mixture, which is slow and only very limited. Therefore, unfortunately, the exact amount of dissolved salt is unknown. For a quantitative comparison with the simulations, hence a fitting procedure is applied (Supplementary Note 1).

Within 3 h after mixing, the samples are transferred to a home-built sample cell^62^ and imaged with a confocal unit (Visitech VT-Eye and Nikon A1R-MP for the structural and dynamic measurements, respectively) that is mounted on an inverted microscope (Nikon Ti-U and Nikon Ti-E, respectively) equipped with an oil immersion objective (Nikon Plan Apo VC × 100). In the structural measurements, for each sample, 25–32 image stacks are recorded, each consisting of 151 frames (512 × 512 pixels) with *z* steps of 200 nm, corresponding to a volume of 54 × 54 × 30 μm^3^, which contains about 7,000 particles. With a rate of 30 slices per second and averaging of 3 slices to obtain 1 frame, a sampling time smaller than 20 s per stack is achieved. For the determination of the dynamics, two-dimensional slices (512 × 512 pixels) corresponding to an area 63.5 × 63.5 μm^2^ are recorded. For each sample, at least two series with 10,000 slices at a rate of 15 slices per second and 4,000 slices at 1 slice per second are taken.

The particle coordinates are extracted using standard methods^63^ and, where necessary, are refined^62^. The mean squared displacement is calculated from the particle coordinates according to 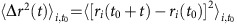 where the average is taken over particles *i* and starting times *t*_0_. For the intermediate scattering function 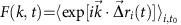

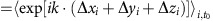
 the magnitude of the scattering vector is chosen as *k*=2*πρ*^1/3^≈2.37 μm^−1^. The delay time is scaled with the Brownian time 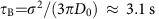 for our system with the short-time self-diffusion coefficient *D*_0_=*k*_B_*T*/*γ* and the friction coefficient of a particle, γ. Note that we correct for possible drift in the set-up by subtracting the centre-of-mass motion of all particles in the observation volume.

### Computer simulations

We simulate a system consisting of *M* particles with mean diameter *σ*_0_ and a polydispersity of about 7%. The polydispersity is realized by shifting the interaction potentials multiple times corresponding to 17 different particle diameters *σ*_*i*_, whose numbers *M*_*i*_ are normally distributed with a standard deviation of the diameter of 0.07*σ* and 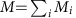. If not stated otherwise, *M*=9,856.

Particles interact with a potential *V*(*r*_*ij*_)=*V*_C_(*r*_*ij*_)+*V*_D_(*r*_*ij*_) that is composed of a repulsive screened Coulomb part^64^


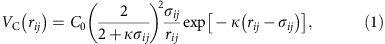


where *σ*_*ij*_=(*σ*_*i*_+*σ*_*j*_)/2, and a short-ranged depletion potential according to the continuous Asakura–Oosawa potential^34^,^65^ plus a steep power law potential


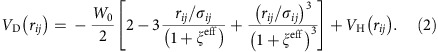


Motivated by the experiments, *ξ*^eff^=0.03 is fixed although in our model this does not necessarily imply that the position of the potential minimum is fixed. The hard interaction of particles at contact is approximated by 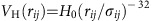 (ref. 66). The prefactor *W*_0_ defines the energy scale of the attractive part and the prefactor *H*_0_ the energy scale of the hard-sphere-like potential and is kept constant at *H*_0_=0.25 *k*_B_*T*. We cut the potential and the corresponding force at 4*σ* and shift them to zero at the cut-off distance. In our model, the range of the attractive part in the potential may vary while other parameters are changed. The interaction potential exhibits a minimum 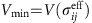 with the effective diameter 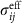 slightly smaller than *σ*_*ij*_. The effective packing fraction 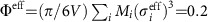, with *V* the volume of the system, is kept constant. For bulk simulations we employ periodic boundary conditions to the cubic simulation boxes. For simulations with parallel flat walls, particles are exposed to an external force, which is defined for the bottom wall as


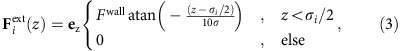


where we fix *F*^wall^*σ*=10^4^*k*_B_*T*. An analogous force is introduced for the top wall. These forces imply interactions close to hard wall interactions but with a small soft contribution to be suitable for simulations. In our Brownian dynamics simulations particle trajectories are governed by their individual Langevin equations^67^


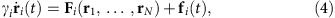


with the friction coefficient *γ*_*i*_ and 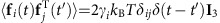, where 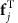 is the transpose of **f**_*j*_, **I**_3_ the three-dimensional unity matrix, *γ*_*i*_ the friction coefficient of a particle with diameter *σ*_*i*_, and *δ*(*t*−*t*′) and *δ*_*ij*_ the Dirac delta and the Kronecker delta, respectively.

As in the experiments, the Brownian time 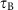
=*σ*^2^/(3*πD*_0_), with the short-time self-diffusion coefficient *D*_0_=*k*_B_*T*/*γ* and the friction coefficient of a particle with average diameter, *γ*, is used as time scale. The time steps are 10^−4^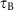 or less. Starting from an initially randomly distributed ensemble, the systems are equilibrated for 300 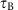 before statistics are gathered, unless stated otherwise.

### Data availability

The data from experiments as well as the data of the simulations are available from the corresponding author.

## Additional information

**How to cite this article:** Kohl, M. *et al*. Directed percolation identified as equilibrium pre-transition towards non-equilibrium arrested gel states. *Nat. Commun.* 7:11817 doi: 10.1038/ncomms11817 (2016).

## Supplementary Material

Supplementary Information Supplementary Figure 1, Supplementary Table 1, Supplementary Notes 1-2 and Supplementary References.
