# Supplementary material for: Directed percolation identified as equilibrium pre-transition towards non-equilibrium arrested gel states
Source: Nat Commun. 2016 Jun 9;7:11817. doi: 10.1038/ncomms11817 (PMC4906224; doi:10.1038/ncomms11817)
Supplement: Supplementary Information — Supplementary Figure 1, Supplementary Table 1, Supplementary Notes 1-2 and Supplementary References. [file ncomms11817-s1.pdf]

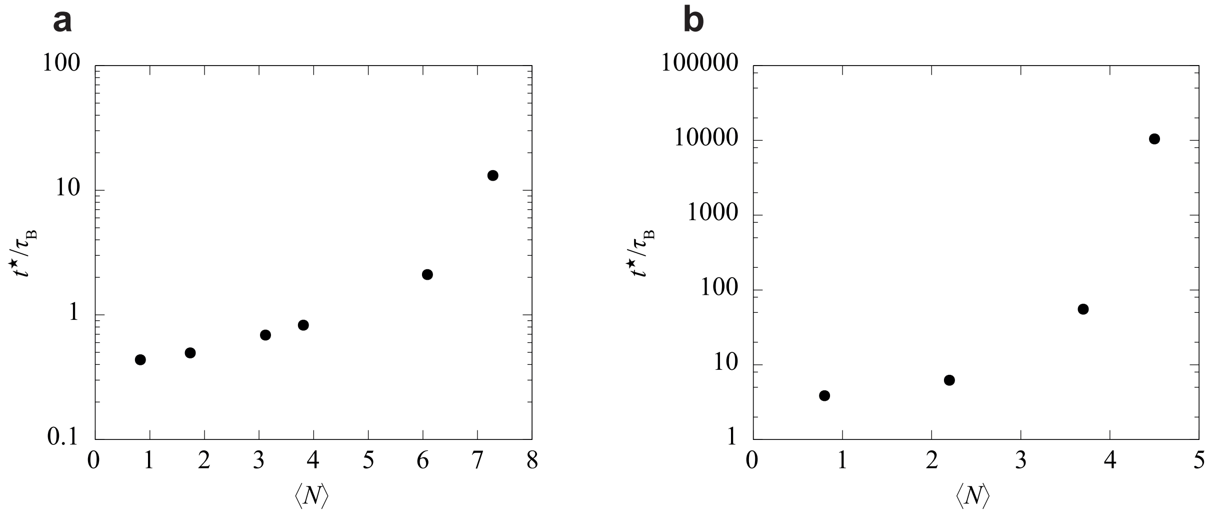

Supplementary Figure 1: Characteristic decay time of the self intermediate scattering function. Characteristic decay time $t^\star$ at which the self intermediate scattering function $F(k,t)$ reaches 0.4, normalized by the Brownian time τ_B, as a function of the mean number of bonds per particle $\langle N \rangle$ as obtained by (a) simulations and (b) experiments. The times $t^\star$ have been determined by fitting a single exponential decay to $F(k,t)$ at short times (Fig. 5).

Sample	Φ	c_p/c_p^*	c_p^{free}/c_p^*	ξ^{eff}	c_{salt} [mM]
B1	0.19	1.46 ± 0.01	1.95 ± 0.01	0.031 ± 0.001	3.5 ± 0.5
B2	0.23	1.54 ± 0.01	2.06 ± 0.01	0.030 ± 0.001	9.4 ± 0.5
B3	0.21	1.55 ± 0.01	2.01 ± 0.01	0.030 ± 0.001	20.1 ± 0.5
C1	0.20	2.25 ± 0.01	2.87 ± 0.01	0.025 ± 0.001	0.0 ± 0.5
C2	0.23	2.24 ± 0.01	2.98 ± 0.01	0.024 ± 0.001	3.2 ± 0.5
C3	0.20	2.25 ± 0.01	2.87 ± 0.01	0.025 ± 0.001	4.0 ± 0.5
C4	0.20	2.24 ± 0.01	2.86 ± 0.01	0.025 ± 0.001	7.8 ± 0.5
C5	0.24	2.24 ± 0.01	3.02 ± 0.01	0.024 ± 0.001	8.4 ± 0.5
D1	0.19	0.76 ± 0.01	0.97 ± 0.01	0.045 ± 0.001	2.8 ± 0.5

Supplementary Table 1: Experimental samples and their compositions. Colloid volume fraction Φ; bulk polymer concentration normalized by the overlap concentration, c_p/c_p^*; free polymer concentration, i.e. in the volume not occupied by the colloids[1–3], normalized by the overlap concentration, c_p^{free}/c_p^*; effective polymer-colloid size ratio ξ^{eff}; salt concentration c_{salt}.

Supplementary Notes

Supplementary Note 1:
Mapping of simulation and experimental parameters

Two aspects are impeding a direct calculation of the interaction potential (Eqs. 1, 2) from the experimental values of the polymer, c_{p}, and salt, c_{salt}, concentrations. The Asakura-Oosawa potential is not quantitatively accurate at the large polymer concentrations and, furthermore, although c_{salt} is well-known experimentally, the amount of salt that is effectively dissolved and/or the conductivity, and hence the ionic strength, cannot be determined accurately enough[4]. The low dielectric constant of the organic solvent mixture also precludes an exact measurement of the zeta potential of the particles[5,6]. Due to these uncertainties, a fitting procedure has been applied to determine the best parameter values of the potential (Eqs. 1, 2), which is guided by the experimentally determined pair correlation functions $g(r)$ (Fig. 1b). This is done following two procedures. First, all parameters are fitted, which provides very good fits to $g(r)$ (Fig. 1b, light blue line) but unreasonable values for C_0, κ and W_0, as explained and discussed in the last paragraph of this Supplementary Note 1. Hence this procedure is not used to obtain the diagram in Fig. 2 but the results are reported for comparison (Figs. 1b, 3c inset). Second, the parameters of the depletion (electrostatic) potential are kept constant along path B (path A) and the knowledge on the sequence of increasing strength and range of the electrostatic (depletion) potential is exploited. (Details are explained in the following.) This yields reasonable values for C_0, κ and W_0, but compromises the quality of the fits somehow. Nevertheless, this procedure is applied to obtain Fig. 2.

Calibration of $\mathbf{C_0}$. The experiments have been carried out with charged particles. The charge of the particles is contained in the interaction parameter C_0 in the electrostatic part of the interaction potential (Eq. 1). Using simulations, C_0 is fitted. The fit is based on the pair correlation function $g(r)$ of a sample without added salt (C1, see Supplementary Table 1) and thus interactions that are dominated by the electrostatic interaction. In the fit, only the parameter C_0 (Eq. 1) is varied and, as suggested by the low salt concentration, $\kappa\sigma = 0.25$ is fixed. Best agreement with the data was obtained for $C_0 = 200\, k_{\text{B}}T$. Consequently, this value was used for all samples. With C_0 fixed, the attraction strength W_0 (Eq. 2) was successively increased.

Fitting based on the mean number of bonds $\langle \mathbf{N} \rangle$. In order to map the experimental samples onto the $V_{\text{D,min}}$– $\kappa\sigma$ plane (Fig. 2), the positions of all samples have been fitted simultaneously based on their mean number of bonds $\langle N \rangle$ and taking into account the following two constraints: First, their relative distances along the $\kappa\sigma$ axis is given by their relative rather than their absolute screening lengths according to Supplementary Table 1. Second, the attraction strengths W_0 corresponding to the three polymer concentrations $c_{\text{p}}^{\text{free}} = [c_{\text{p}}^*, 2c_{\text{p}}^*, 3c_{\text{p}}^*]$ are assumed to differ by at least $4\, k_{\text{B}}T$. Thus, only affine transformations of the sample positions in the $V_{\text{D,min}}$– $\kappa\sigma$ plane are considered in a simultaneous fit of all sample positions. The absolute positions were determined by minimizing the difference between $\langle N \rangle_{\text{sim}}$ and $\langle N \rangle_{\text{exp}}$ as determined in simulations and experiments, respectively, i.e. the minimum $\text{Min}\{\sum_{\text{B}_i,\text{C}_i,\text{D}_i} |\langle N_j \rangle_{\text{sim}} - \langle N_j \rangle_{\text{exp}}|\}$, where the individual $\langle N_j \rangle_{\text{sim}}$ are constrained by the allowed affine transformation, as explained above. This fit determines the relative positions of all samples, such that the overall agreement between the mean

number of bonds from experiments and simulations are optimized. While this fitting procedure is appropriate for our purposes, there might be small deviations to the precise absolute positions of the experimental samples.

Unconstrained fit. In order to further optimize the individual sample positions within the $V_{\mathrm{D,min}}$– $\kappa\sigma$ plane, we additionally performed a free fit where the parameters are not constrained to any path. This fit is based on the minimization of the absolute error vector $\Delta g(r) = |g_{\mathrm{sim}}(r) - g_{\mathrm{exp}}(r)|$ between the pair correlations in simulations and experiments, respectively, over the interval $[\sigma, 3\sigma]$. Thus, the pair correlation functions, $g(r)$ (Fig. 1b), and the distributions of the angle between two successive bonds, $p(\varphi)$ (Fig. 3c, inset), agree better, but the fitted positions , i.e. $V_{\mathrm{D,min}}$ and κ, are not fully consistent with the compositions of the samples.

Supplementary Note 2:
Characteristic decay time $t^\star$ of the self intermediate scattering function $F(k,t)$ in directed percolated systems

In directed percolated systems the dynamics of the system slows down significantly, which is quantified by the self intermediate scattering function $F(k,t)$ (Fig. 5). The characteristic decay time $t^\star$ of $F(k,t)$ is defined as the time required to reach $F(k,t^\star) = 0.4$. Not all $F(k,t)$ decay to 0.4 in the time windows of the experiments. Thus, a single exponential decay was fitted to $F(k,t)$ at short times with $F(k,0)$ set to the value of the first data point and $F(k,\infty)$ set to 0. This leaves only the characteristic decay time $t^\star$ as free parameter. For consistency, this fitting procedures was applied to simulation and experimental data sets and both data sets are represented as a function of the mean number of bonds per particle $\langle N \rangle$.

The dependencies of $t^\star$ on $\langle N \rangle$ are presented in Supplementary Fig. 1. In simulations as well as in experiments, $t^\star$ increases moderately for small $\langle N \rangle$, but increases by several orders of magnitude for large $\langle N \rangle$ that correspond to directed percolation. This trend is consistently observed in simulations and experiments. Note that the experimental and simulation results cannot be compared directly, because they are determined along different paths.

Supplementary References

[1] Fleer, G. J. & Tuinier, R. Analytical phase diagram for colloid-polymer mixtures. *Phys. Rev. E* **76**, 041802 (2007).

[2] Lekkerkerker, H. N. W., Poon, W. C. K., Pusey, P. N., Stroobants, A. & Warren, P. B. Phase behaviour of colloid-polymer mixtures. *Europhys. Lett.* **20**, 559–564 (1992).

[3] Aarts, D. G. A. L., Tuinier, R. & Lekkerkerker, H. N. W. Phase behaviour of mixtures of colloidal spheres and excluded-volume polymer chains. *J. Phys.: Condens. Mat.* **14**, 7551–7561 (2002).

[4] Dukhin, A. & Parlia, S. Ions, ion pairs and inverse micelles in non-polar media. *Curr. Opin. Coll. Interf. Sci.* **18**, 93–115 (2013).

[5] Miller, J. F., Schätzel, K. & Vincent, B. The determination of very small electrophoretic mobilities in polar and nonpolar colloidal dispersions using phase analysis light scattering. *J. Coll. Interf. Sci.* **143**, 532–554 (1991).

[6] McNeil-Watson, F., Tscharnuter, W. & Miller, J. A new instrument for the measurement of very small electrophoretic mobilities using phase analysis light scattering (PALS). *Colloids Surf. A Physicochem. Eng. Asp.* **140**, 53–57 (1998).
